# OASIS: Online Application for the Survival Analysis of Lifespan Assays Performed in Aging Research

**DOI:** 10.1371/journal.pone.0023525

**Published:** 2011-08-15

**Authors:** Jae-Seong Yang, Hyun-Jun Nam, Mihwa Seo, Seong Kyu Han, Yonghwan Choi, Hong Gil Nam, Seung-Jae Lee, Sanguk Kim

**Affiliations:** 1 School of Interdisciplinary Bioscience and Bioengineering, Pohang University of Science and Technology, Pohang, South Korea; 2 Department of Molecular Life Science, Pohang University of Science and Technology, Pohang, South Korea; 3 World Class University Information Technology Convergence Engineering, Pohang University of Science and Technology, Pohang, South Korea; Universita' del Piemonte Orientale, Italy

## Abstract

**Background:**

Aging is a fundamental biological process. Characterization of genetic and environmental factors that influence lifespan is a crucial step toward understanding the mechanisms of aging at the organism level. To capture the different effects of genetic and environmental factors on lifespan, appropriate statistical analyses are needed.

**Methodology/Principal Findings:**

We developed an online application for survival analysis (OASIS) that helps conduct various novel statistical tasks involved in analyzing survival data in a user-friendly manner. OASIS provides standard survival analysis results including *Kaplan-Meier* estimates and mean/median survival time by taking censored survival data. OASIS also provides various statistical tests including comparison of mean survival time, overall survival curve, and survival rate at specific time point. To visualize survival data, OASIS generates survival and log cumulative hazard plots that enable researchers to easily interpret their experimental results. Furthermore, we provide statistical methods that can analyze variances among survival datasets. In addition, users can analyze proportional effects of risk factors on survival.

**Conclusions/Significance:**

OASIS provides a platform that is essential to facilitate efficient statistical analyses of survival data in the field of aging research. Web application and a detailed description of algorithms are accessible from http://sbi.postech.ac.kr/oasis.

## Introduction

During the last two decades, we have witnessed the explosion of the field of aging research. For identifying mechanisms of aging, many approaches have been attempted to discover genetic and environmental factors that regulate aging in various organisms [1], [2]. A key experiment for examining the effects of genetic modulation or chemical compounds on aging is the measurement of lifespan, which requires analysis of survival over time during aging processes. By performing appropriate statistical analyses on survival data, one can extract a wealth of useful information (Table S1) [3]–[6]. For example, a log-rank (Mantel-Cox) test was introduced to determine whether experimental treatments significantly affected lifespan or not [7], [8]. In addition, analysis of hazard function from lifespan data has gained popularity because the shape of the cumulative hazard plots has been proposed to reflect the rate of aging [9]. Therefore, accurate and efficient execution of statistical analyses is a crucial step towards a better understanding of aging at the molecular level.

Despite the development of statistical analyses of lifespan data, there is a need for developing further statistical methods to explain complex phenomena involved in aging. One of the interesting characteristics of aging is that even relatively homogeneous individuals under controlled environmental conditions often display variations in lifespan [10], [11]. That is, some populations in a mostly homogeneous genetic background show precipitous survival curve at a specific time point whereas others display gradual survival curve. One possible explanation for this phenomenon is that stochastic components such as epigenetic switch or noisy gene expression, which may be influenced by some unknown factors, play an important role in this variation in lifespan. In addition, genetic components have been suggested to contribute the variances in lifespan [12]. Analyzing the contribution of such factors will require a novel statistical test that can quantify the variances of lifespan data.

Here we report an open-access service for survival analysis, the online application for survival analysis (OASIS) which provides not only canonical survival analysis methods but also advanced statistical tests for comparing the variances in survival datasets. OASIS is a user-friendly online application which runs in a browser without downloading or installation. These features of OASIS will not only help researchers in the field of aging research analyze their data in depth but will potentially facilitate the standardization of survival analysis.

## Results

### OASIS web application

**Figure 1:**
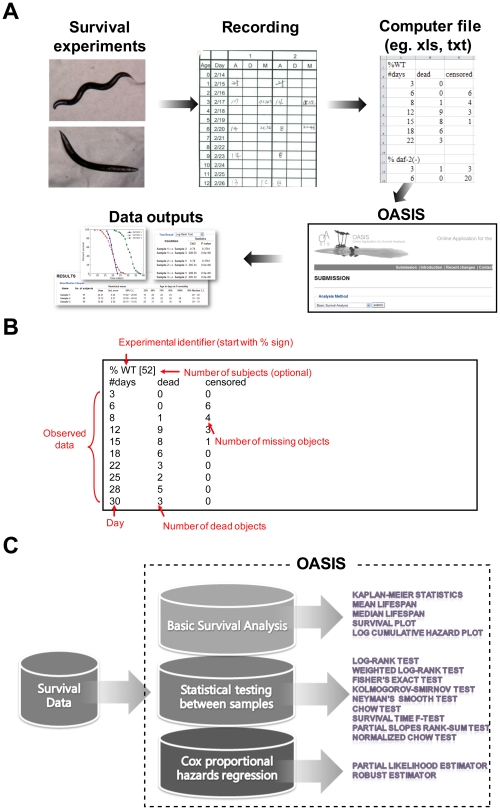
Statistical components of the OASIS. (A) Overall flowchart of survival analysis (B) Input for basic survival analysis and statistical testing. Observed data have at least three columns; time after observation started, the number of dead subjects, and the number of censored subjects during the interval. (C) The web application provides a uniform platform that comprises of three analysis parts: basic survival analysis, statistical testing, and Cox proportional hazards regression.

To provide a standardized platform for biologists in aging research fields to perform survival analyses, we developed OASIS server which is accessible by using the majority of modern web browsers. After surveying experiments, recorded survival data are imported as an input of OASIS web server in a simple format (Figure 1A). The input format should consist of following items in a given order: an experimental identifier and observed data. The line started with “%” sign indicates the experimental identifier. Observed data have at least three columns: observed time, the number of dead subjects, and the number of censored (e.g. missing) subjects during the observation interval (Figure 1B; sample inputs are available in the OASIS webpage). These columns should be separated with tabs.

**Figure 2:**
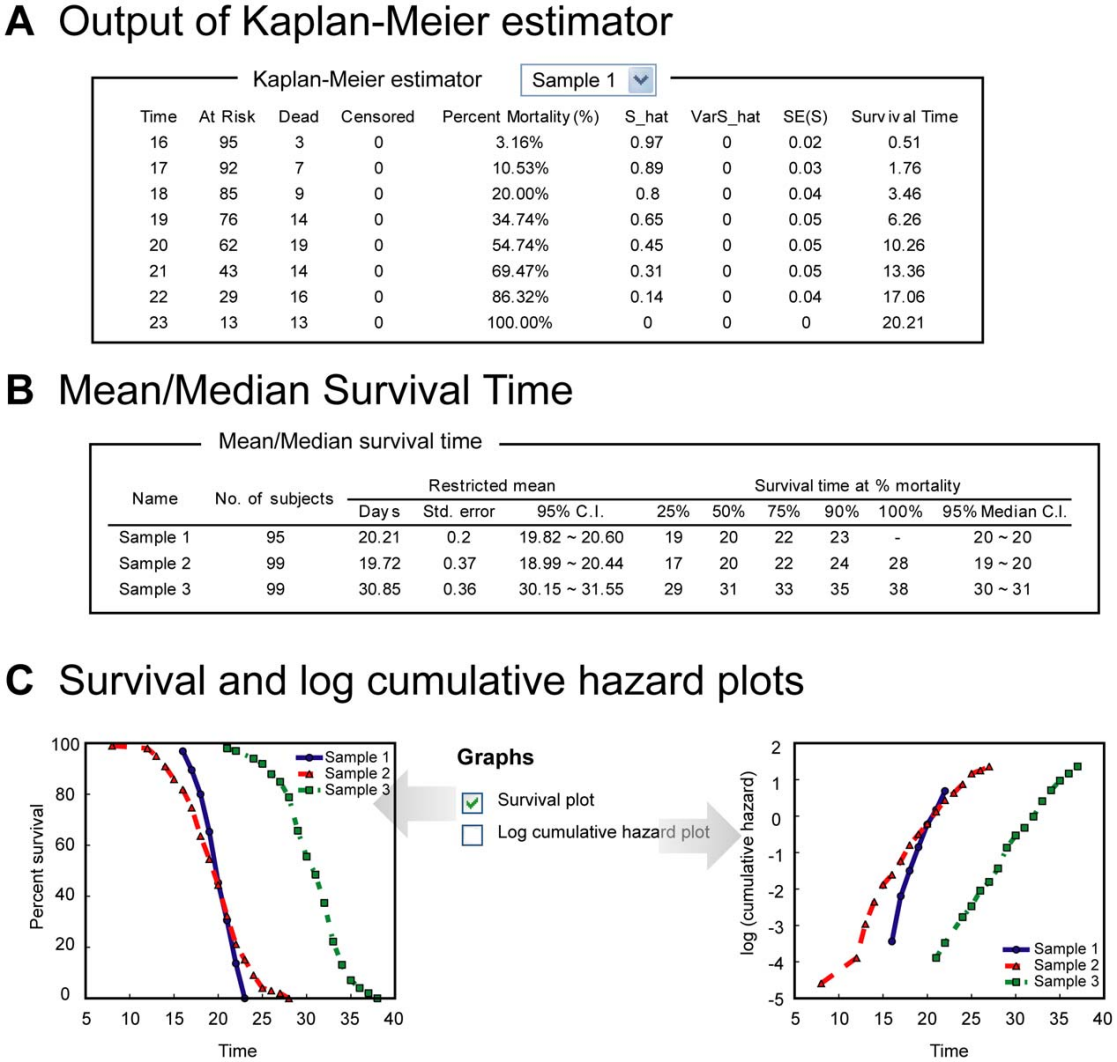
Results of basic survival analysis. (A) The output of Kaplan-Meier estimator. (B) Mean/median survival time of data. Survival time at 25%, 50%, 75%, 90%, and 100% mortality are shown. (C) Survival and log cumulative hazard plots.

**Figure 3:**
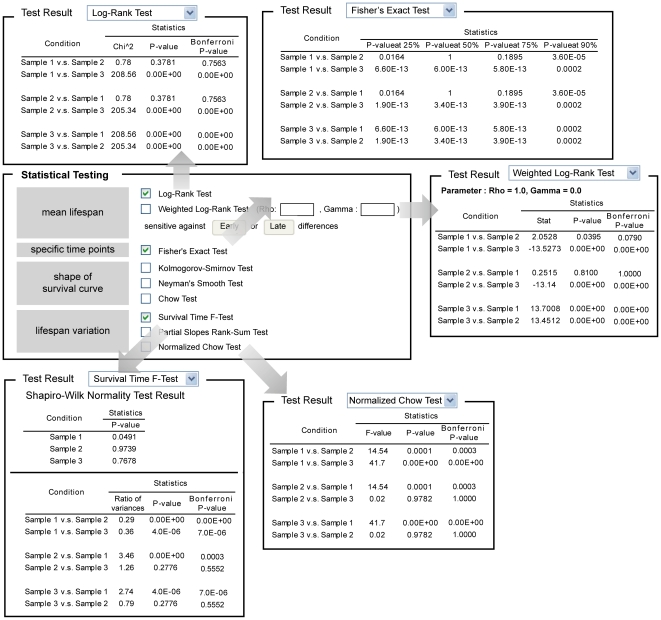
Results of statistical testing and input format. Output of statistical tests for differences in lifespan data between samples.

**Figure 4:**
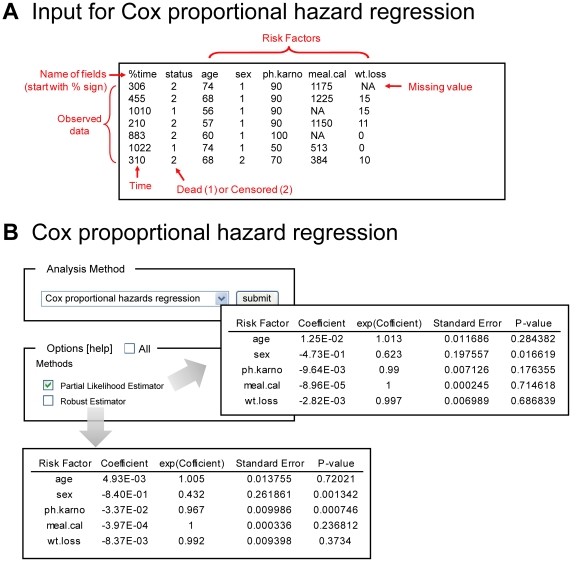
Results of the Cox proportional hazard regression and input format. (A) Input for Cox proportional hazard regression. Observed data have at least three columns; observed time, the status of events (dead or censored), and the values of risk factors. (B) Output of Cox proportional hazard regression.

OASIS is composed of three statistical frameworks that can facilitate proper analysis of survival data (Figure 1C). In the basic survival analysis, OASIS provides various statistical methods such as Kaplan-Meier statistics, mean and median survival time, survival and log cumulative hazard plots for depicting the characteristics of each dataset (Figure 2). For statistical comparisons of different survival datasets, OASIS performs hypothesis testing such as log-rank test, Fisher's exact test, Kolmogorov-Smirnov test, and Neyman's smooth test (Figure 3). In particular, survival time *F*-test, partial slopes rank-sum test, and normalized chow test are novel testing methods for the comparison of the shape of survival or hazard functions. Furthermore, OASIS generates hazards regression that can evaluate the effect of several risk factors (Figure 4). By using these outputs produced by OASIS, users can interpret survival data comprehensively.

### Statistical testing for differences in the length of lifespan

A comprehensive comparison of survival datasets between an experimental group and a control group is important to determine the effects of experimental treatments on survival. For example, some drug treatments can only increase the average survival time, whereas others can increase both average and maximum survival times. Therefore, to distinguish these differences OASIS provides various statistical comparison methods (Figure 3). For example, the statistics of average survival time can be obtained by using log-rank test, whereas those of a specific time point can be obtained by using Fisher's exact test [13]. OASIS provides log-rank test, Fisher's exact test, and other tests that are explained in the followings.

#### 1.1 Log-rank test

Mantel-Cox test, so-called log-rank test, is a kind of nonparametric test that is frequently used for comparing two survival functions through overall lifespan data [7], [8], [14]–[17]. The log-rank statistics in two groups such as an experimental and a control groups is calculated as follows.



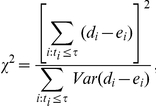


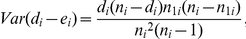
where *d_i_* is the number of deaths in group 1, and *e_i_* (estimated as 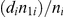) is the number of expected deaths in group 1. *n_1i_* is the size of the population of group 1 at risk during the *i^th^* interval, and *n_i_* is the total size of population at risk during the *i^th^* interval.

#### 1.2 Fisher's exact test

Fisher's exact test is frequently used in survival analysis [6], [13], [18]–[24]. To test different survival functions at specific time points instead of overall lifespan, the program can calculate the probability of observed data with Fisher's exact test at different time points using the following formula.



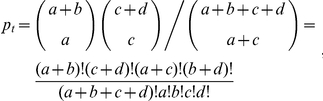
where *a* and *b* are the numbers of living subjects in group 1 and group 2 respectively and *c* and *d* are that of dead subjects in group 1 and group 2 respectively at the specific time *t*. *P*-value of Fisher's exact test is calculated as the sum of probabilities less than or equal to *p_t_* of all combinations. Generally, 90% mortality rate is used for Fisher's exact test. However, in some cases, comparisons between two datasets at 90% mortality show no statistically significant difference because of several reasons including drastic death at an old age. This suggests that one might want to put more emphasis on earlier deaths than later ones because later deaths might result from causes unrelated to normal aging.

#### 1.3 Weighted log-rank test

As mentioned in the previous paragraph, one might want to put more emphasis on earlier deaths than the later ones or vice versa. To generalize log-rank test for these needs, Fleming and Harrington developed *G(rho, gamma)-*weighted log-rank test [25], [26]. The weighted test statistics is calculated by the following equation.



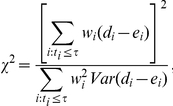
where *w_i_* represents the *G*(*rho*, *gamma*) weight defined as *S*(*t*)*^rho^*(*1-S*(*t*))*^gamma^*. Generally if rho >0 and gamma  = 0, the test is sensitive to early differences, whereas if rho  = 0 and gamma >0, the test is sensitive to later differences [26].

### Statistical testing for differences in the shape of hazard function

#### 2.1 Kolmogorov-Smirnov test

While the log-rank test is commonly used for comparing survival data between samples, it is optimized for special assumptions on the underlying distributions such that the hazard ratio or relative risk λ_2_(*t*) / λ_1_(*t*) is constant in time *t*. In that case, a log-rank test generally gives optimal results. However, one may need statistical tests that do not depend on the distribution of survival data. The Kolmogorov-Smirnov test is suitable for this purpose so that it robustly works in the condition where the hazard functions λ_1_(*t*) and λ_2_(*t*) cross over through time *t*. The Kolmogorov-Smirnov test is based on the following equation.



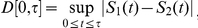
where sup represents a supremum of a set that gives the smallest real number that is greater than or equal to every number in the set and *D* represents the largest absolute vertical deviation. OASIS adopted *surv2.ks* function implemented in the R packages [27] to provide Kolmogorov-Smirnov test. We note that the Kolmogorov-Smirnov test in OASIS is not applicable to survival data that contain tied observations [e.g. multiple events (deaths or failures) during an observed time interval]. OASIS provides a warning message if there is any tied observation in survival data within or between samples.

#### 2.2 Neyman's smooth test

Another statistical comparison method for detecting a wide spectrum of alternatives is Neyman's smooth test. It was developed to test the homogeneity of two different survival data by comparing a null model, *S*_1_(*t*) = *S*_2_(*t*) to various alternative models. The alternative models embedded the null model with Legendre polynomials based on Neyman's goodness-of-fit idea as the following equation.



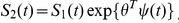
where *θ*  = (*θ*_1_,…, *θ*_d_)^T^ is a parameter set of bounded functions *ψ* (*t*) = (*ψ*_1_(*t*), …, *ψ*_d_(*t*))^T^, which models possible differences between *S*_1_(*t*) and *S*_2_(*t*). Therefore, if *θ*  = 0, null hypothesis is accepted. Since the Neyman's smooth test selects optimal smooth model in Legendre polynomials with Schwarz's selection rule, it is different from Kolmogorov-Smirnov test with respect to providing an idea of the types of difference between two survival data [28]. The selected dimension represents a type of difference between *S*_1_(*t*) and *S*_2_(*t*). If the selected dimension (*d*) is 1, this suggests that *S*_1_(*t*) is different from *S*_2_(*t*) by the constant hazard ratio. If the selected *d* is 2, the relationship between two samples is likely to be monotonic. If the selected *d* is 3, the relationship between two samples is likely to have a convex or a concave form. OASIS adopted *surv2.neyman* function implemented in the R packages [28] to provide the Neyman's smooth test. Similar to the Kolmogorov-Smirnov test, the Neyman's smooth test is not currently applicable when there are tied observations in survival data.

#### 2.3 Chow test

Chow test, a variant of *F*-test, was invented by economist Gregory Chow to test whether the coefficients in two linear regressions on different data sets are same or not [29]. This test is generally used for detecting structural break that is an unexpected shift in time series data. In OASIS, we used this analysis for detecting structural differences between two different log cumulative hazard functions by using the following equation.



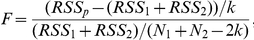
where *RSS_p_* represents the sum of squared residuals from pooled log cumulative hazard data. *RSS_1_* and *RSS_2_* represent the sum of squared residuals from two different log cumulative hazard data respectively. *N_1_* and *N_2_* are the numbers of observation in each data and *k*, which is 3 in this case, is the total number of parameters of linear regression model. The test statistic follows the *F* distribution with (*k*, *N_1_* + *N_2_* – *2k*) degrees of freedom.

### Statistical testing for differences of the variances in survival time

#### 3.1 Survival time *F*-test

We provide survival time *F*-test, which is used to examine whether two normal populations have the same variance or not. Because censored data are generally used in survival analysis, one can estimate the number of dead animals using survival function *S(t)* and then perform *F*-test for the comparison of variances of two different survival data. The *F*-test is used under the condition that the survival times of individuals follow a normal distribution. As a normality check method for a given dataset, we provide the Shapiro-Wilk test in the OASIS website. If the *P*-value generated by the Shapiro-Wilk test is smaller than 0.01, then the chance of survival data following the normal distribution is less than 0.01. In that case, we provide a warning message because the results of *F*-test are not applicable.

#### 3.2 Partial slopes rank-sum test

We devised another statistical test method for comparing the differences in the slopes of two log cumulative hazard plots. We calculated partial slopes of the log cumulative hazard plot. With null hypothesis that two different log cumulative hazard plots have same slopes, we conducted rank-sum tests with set of partial slopes as following definitions.



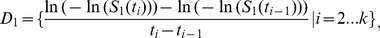


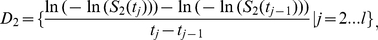
where *D_1_* and *D_2_* are sets of partial slopes of each group. These sets are compared with rank-sum test.

The partial slopes rank-sum test is based on a non-parametric statistics that requires sufficient number of samples (in this case, partial slopes) for the reliable analysis. Since a partial slope is defined as the changes in log cumulative hazard divided by the corresponding change in survival time between two neighbouring time points, the number of observed time points rather than the total sample size is important for this non-parametric analysis. To obtain statistically meaningful results, at least six observed time points are needed.

#### 3.3 Normalized chow test

Chow test is used for testing whether the coefficients in two linear regressions on different datasets are same or not [29]. However, researchers who perform survival analysis tend to be interested in examining the difference in slope rather than in determining the difference in y-intersect. For this purpose, before conducting Chow test, we normalized the log cumulative hazard data to have a mean of zero. In this case, the linear regression of each dataset has zero y-intersect. Thus, one can examine the differences in the slopes of datasets and pooled data. We verified the difference in the lifespan variations through normalized Chow test, a statistical test that examines whether the coefficients of two linear regressions on different normalized data sets are equal. Similar to the log-rank test, the assumption is that survival rate is constant over time to apply the normalized Chow test.

### Application of basic survival analysis to experiments

**Figure 5:**
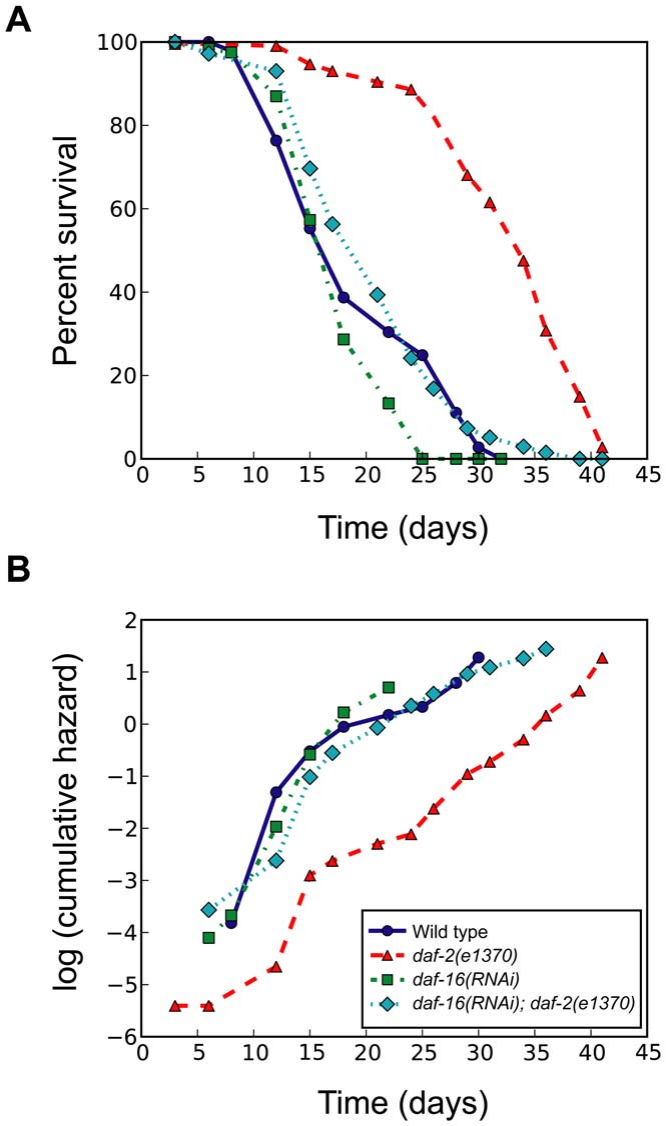
Survival analysis of wild-type and *daf-2* mutant *C. elegans* in combination with *daf-16* RNA interference. (A) Survival plots and (B) log cumulative hazard plots of wild type, *daf-2* mutant [*daf-2(e1370)*], *daf-16(RNAi)*, and *daf-2* mutant treated with *daf-16* RNAi [*daf-16(RNAi); daf-2(e1370)*] animals.

As a test case, we analyzed a lifespan dataset of the roundworm *C. elegans* using OASIS. Mutations in *daf-2*, which encodes an insulin/IGF-1 receptor homolog, extend the lifespan of *C. elegans* [30], [31]. The FOXO transcription factor DAF-16 is required for *daf-2* mutants to live long [32]-[35]. We measured the lifespan of wild-type and *daf-2(e1370)* mutant animals with or without knocking down the *daf-16* transcription factors using RNA interference. OASIS provided the result that *daf-2(e1370)* mutation significantly extended lifespan and decreased mortality rate (Figure 5). By using OASIS, we generated the survival and log cumulative hazard plots that illustrated these differences (Figure 5). Among the groups of our experiments, only *daf-2(e1370)* mutants showed a different survival pattern from others using these graphs. The survival curve of *daf-2(e1370)* mutants was shifted to the right compared to others (Figure 5A). In addition, the y-intercept in the log cumulative hazard plot of *daf-2(e1370)* mutants was smaller than those of others (Figure 5B). To statistically validate these results that *daf-2(e1370)* mutants live long, we compared the mean lifespan and conducted various statistical tests. The mean lifespan of *daf-2(e1370)* mutants is larger than 30 days, whereas those of other groups are approximately 20 days (Table 1). By using log-rank test and Fisher's exact test, we determined the statistical significance of these lifespan differences. By using log-rank test, we showed that the wild type and *daf-2(e1370)* have significantly different mean lifespans (*P*<1.0×10^−10^), whereas the wild type and *daf-2(e1370)* mutants treated with *daf-16* RNAi have similar mean lifespans (*P* = 0.113). In addition, proportions of survivors at specific time points can be compared by using Fisher's exact tests. These statistical analyses acquired by using OASIS are consistent with previous reports [30]–[35]. Furthermore, we examined the OASIS analysis by comparing with publicly available datasets and confirmed its validity (Table S2).

**Table 1:** Statistical analysis for the lifespan data of *daf-2* mutants in combination with *daf-16* RNAi knock down.

Statistics									
Name	No. of subjects	Restricted mean			Age in days at % mortality (days)				
		Days	Std. Err.	95% C.I.	25%	50%	75%	90%	100%
Wild type	52	19.35	1.07	17.24∼21.45	15	18	25	30	32
*daf-2(e1370)*	223	32.87	0.64	31.63∼34.12	29	34	39	41	-
*daf-16(RNAi)*	127	17.74	0.43	16.91∼18.57	15	18	22	25	28
*daf-16(RNAi); daf-2(e1370)*	366	20.84	0.46	19.94∼21.73	15	21	24	29	39

Comparison between samples							
Type	Name	Log-rank test		Fisher exact test at % mortality (*P*-value)			
		χ^2^	Prob > χ^2^	25%	50%	75%	90%
Control	Wild type						
	*daf-2(e1370)*	119.2	0.00E+00*	4.04E-11	1.09E-13	7.53E-07	9.35E-05
	*daf-16(RNAi)*	5.03	2.50E-02	1.00E+00	2.52E-01	6.03E-02	3.69E-03
	*daf-16(RNAi); daf-2(e1370)*	2.51	1.13E-01	5.55E-02	4.15E-01	1.83E-01	2.59E-02
Control	*daf-2(e1370)*						
	Wild type	119.2	0.00E+00*	4.04E-11	1.09E-13	7.53E-07	9.35E-05
	*daf-16(RNAi)*	206.8	0.00E+00*	1.16E-13	1.10E-12	8.49E-13	1.06E-10
	*daf-16(RNAi); daf-2(e1370)*	146.1	0.00E+00*	2.69E-10	9.56E-13	1.60E-12	5.06E-02

Users can compare restricted mean and “maximum survival time,” which is generally the 90^th^ percentile of survival. C.I. indicates confidence interval. In the case of *daf-2(e1370)* the age in days at 100% mortality (days) was not determined “-”, because the last individual *C. elegans* that survived was censored. * The log-rank test in OASIS provides ‘0.00E+00’ when *P*<1.0×10^−10^.

### Advanced statistical analyses for the comparison of variances in survival data

**Figure 6:**
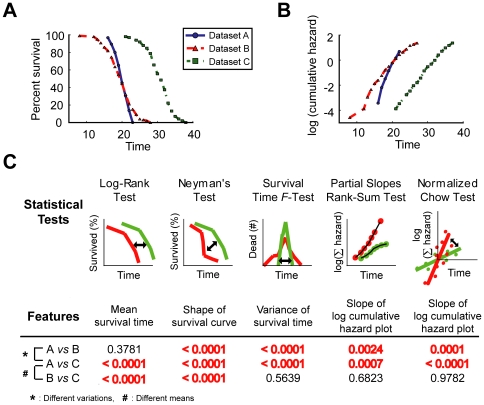
Advanced statistical results analyzing the differences of survival variations using hypothetical datasets. (A) Survival plots and (B) log cumulative hazard plots of different types of hypothetical survival data. (C) Concept figures of each statistical test are illustrated. *P*-values of each statistical test are shown. Significant differences are indicated as bold font in red.

To examine the usefulness of advanced statistical methods in OASIS, we conducted a model-based test to show how different statistical methods implemented in OASIS work on complex survival variations that are beyond the reach of conventional analysis. Three different types of hypothetical survival datasets, A, B and C were generated for developing this test (Figure 6). Datasets A and B have the same mean lifespans (20 days) but different lifespan variances (2 days for A; 4 days for B), whereas dataset B and C have the same lifespan variances (4 days) but different mean lifespans (20 days for B; 30 days for C). The characteristics of each dataset were depicted in the survival (Figure 6A) and log cumulative hazard plots (Figure 6B) where the differences of lifespan variations were not evident. It is difficult to distinguish lifespan variations using conventional methods for survival analysis. Specifically, log-rank test was suitable for the comparison of mean lifespan, whereas Neyman's test was effective to distinguish structural differences between two survival curves (Figure 6C). In contrast, three statistical methods that we implemented in OASIS, the survival time *F*-test, partial slopes rank-sum test, and normalized Chow test, were able to discriminate the variances in the lifespan data (Figure 6C).

**Figure 7:**
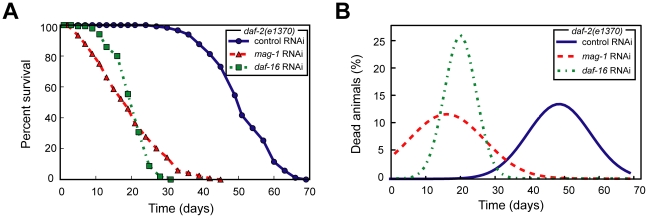
Examples of lifespan variations among experimental datasets. (A) Survival plots of *daf-2(e1370)* mutants treated with control RNAi, *mag-1* RNAi, and *daf-16* RNAi. (B) Gaussian fitting curves for the survival time *F*-test that analyzes the variances of *daf-2(e1370)* mutants treated with control RNAi, *mag-1* RNAi, and *daf-16* RNAi.

We then applied these statistical methods to survival experiments with *C. elegans*. We analyzed the lifespan data of *daf-2(e1370)* mutant *C. elegans* treated with RNAi targeting *daf-16*/FOXO or with RNAi targeting *mag-1*, which encodes a *C. elegans* homolog of *Drosophila mago nashi* known to regulate hermaphrodite germ-line sex determination [36]. We found that both *daf-16* RNAi and *mag-1*RNAi in *daf-2(e1370)* mutant background significantly shortened lifespan compared to control RNAi (*P*<1.0×10^−10^, log-rank test) (Figure 7A). We found that *daf-16* RNAi- and *mag-1* RNAi-treated *daf-2(e1370)* mutants have similar mean lifespans (*P* = 0.82, log-rank test). However, the analyses of lifespan variances using OASIS revealed that *mag-1* RNAi resulted in a significantly different variance in lifespan compared with *daf-16* RNAi (Figure 7B, *P*<1.0×10^−10^, survival time *F*-test; *P*<0.05, partial slopes rank-sum test; *P*<1.0×10^−10^, normalized Chow test). As another example, we analyzed the lifespan datasets of wild-type and *tax-2(p671); isp-1(qm150)* double mutant *C. elegans*, which has mutations in *tax-2* [a subunit of a cyclic-nucleotide gated calcium channel] and *isp-1* [an iron-sulfur protein in complex III in the respiratory chain]. In our previous report, we showed that the mean lifespan values of these two lifespan datasets were similar (*P* = 0.12, log-rank test) but the lifespan curves crossed with each other [37]. Using OASIS, we found that the difference of variances in lifespan between these two datasets is statistically significant (*P*<1.0×10^−10^, survival time *F*-test; *P*<0.05, partial slopes rank-sum test; *P*<0.05, normalized Chow test). Together, these analyses indicate that our advanced statistical methods are useful for distinguishing the differences in lifespan variances. Moreover, the results shown here using *C. elegans* mutants or RNAi suggest that genetic components underlie at least in some cases of the lifespan variances.

## Discussion

Rigorous analysis of survival data is crucial for aging research. Several statistical tools for survival analysis are available (Table S3), but for the first time, we provide OASIS for the comprehensive analysis of survival data including generation of *KAPLAN-MEIER* statistics, visualization of survival and log cumulative hazard plots, statistical test of hypothesis, and hazards regression. OASIS is based upon Django [38], python-based web framework, and R statistical environment [27] to integrate essential and advanced statistical features as well as a user-friendly graphical interface for survival analysis.

Historically, comparison of average survival time was predominantly used for the analysis of survival data. However, in many cases of survival data comparisons as shown in our examples (Figure 7), two survival curves with obviously different shapes may have similar mean survival times and therefore researchers may conclude that the differences in the survival datasets are not statistically significant. As described recently [10], even a population of organisms with relatively homogeneous genetic and environmental factors showed gradual lifespan curves instead of sharp precipitated line. Stochastic factors including epigenetic switch and noisy gene expression [10] and/or genetic components [12] may underlie this phenomenon and it will be crucial to identify and to characterize these factors in the future. Here, we provide advanced statistical methods for analyzing the differences in variances among survival datasets and we believe these methods will be useful to objectively quantify the variances based on statistical significance.

For proper survival analysis, OASIS users should consider underlying statistical assumptions. For example, log-rank test is suitable when the baseline survival rate is not changing over time. In addition, three advanced statistical tests, the survival time *F*-test, partial slopes rank-sum test and normalized chow test, are used to identify the differences in lifespan variations based on specific assumptions. In the statistical testing, OASIS automatically generates all pair-wise comparison results, which may increase type I error [39]. To adjust multiple testing, OASIS provides the corrected *P*-values with Bonferroni method, one of the most commonly used correction methods for multiple statistical comparisons. Together with the consideration of these assumptions, performing lifespan experiments in several times independently will give reliable results for distinguishing the differences in lifespan variations.

### Availability and Requirements

The software is available for public use at http://sbi.postech.ac.kr/oasis.

Because researchers' unpublished data are uploaded to the website, OASIS encrypts input data and does not permit access of other users to ensure security and privacy.

## Methods

### Basic survival analysis

To estimate survival time as the area under the survival curve, it is necessary to characterize the survival function which is a probability of death after some specific time *t*.



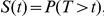
where *t* is some specific time, *T* is a random variable of the time, and *P* denotes the probability of death.

Generally, we only observe censored data that include missing subjects. To consider these missing subjects, the *Kaplan-Meier* estimator was proposed in 1958 [40], [41] for right censored data analysis. The survival function *S(t)* is estimated in the *Kaplan-Meier* method as following formula.



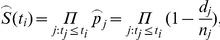
where 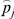
is the conditional probability of survival during the *j^th^* interval, *d_j_* is the number of deaths and *n_j_* is the size of the population at risk during the *j^th^* interval.

Using the *Kaplan-Meier* estimator, OASIS gives various outputs such as variance of survival function, mean and median lifespan, lifespan at % mortality, survival plots, and log-cumulative hazard plots (Figure 2). These outputs generally describe the characteristics of survival data. For example, the slope of log-cumulative hazard plots indicates the age-dependent increase in death rate and the Y-intercept represents the hazard rate at the beginning of the observation. More detailed explanations are in the Text S1.

### Cox proportional hazard regression

Whereas a hypothesis testing is useful for comparing survival data among two or more groups, Cox proportional-hazards regression is suitable for analyzing the proportional effects of several risk factors on survival [42]. OASIS provides Cox proportional hazards regression, which can evaluate the effect of several risk factors such as sex, age, and weight on survival (Figure 4B). Mortality rate can be explained by the proportional sum of risk factors. Cox formulated semi-parametric model with the following equation.
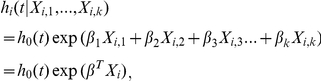
where *X*_i,1_, …, *X*_i,k_ represent *k* risk factors that are assumed to act independently, *β*_1_, …, *β*_k_ are their regression coefficients, *h*_0_(*t*) is the baseline hazard at time *t*, and *i* is a subscript for observation. OASIS provides standard Cox proportional-hazards regression and robust methods [43] (Text S1). Both methods provide regression coefficient of risk factors and their statistical significance.

To identify risk factors that explain hazard function with proportion, the input data format should be different from that of survival analysis. As shown in Figure 4A, OASIS takes following format of input data for Cox proportional hazards regression in the given order; the name of fields and observed data. The line starting with “%” sign indicates the name of fields. Observed data have at least three columns; observed time, the status of events (dead or not), and the values of risk factors. Other columns are considered as the values of risk factors. These columns are separated with tabs.

## Supporting Information

Text S1 **Detailed explanations of statistical methods for survival analyses**
(PDF)

Table S1 **Statistical methods used for survival analyses**
(PDF)

Table S2 **Comparisons of OASIS results with reference data**
(PDF)

Table S3 **Web-based statistical methods for survival analysis**
(PDF)
