# Supplementary material for: OASIS: Online Application for the Survival Analysis of Lifespan Assays Performed in Aging Research
Source: PLoS One. 2011 Aug 15;6(8):e23525. doi: 10.1371/journal.pone.0023525 (PMC3156233; doi:10.1371/journal.pone.0023525)
Supplement: Text S1 — Detailed explanations of statistical methods for survival analyses (PDF) [file pone.0023525.s001.pdf]

OASIS: Online Application for the Survival Analysis of Lifespan Assays Performed in Aging Research

Supplementary Information

Supplementary Methods

Supplementary References

Supplementary Methods

Basic survival analysis

Variance of the survival function

The variance of *Kaplan-Meier* estimator at the time point t_i can be estimated by Greenwood's formula. Although the calculated variance tends to underestimate the true variance of the *Kaplan-Meier* estimator for moderate-sized samples, it is relatively close to the true variance and therefore commonly used in various studies [1].

$$\widehat{V}[\widehat{S}(t_i)] = \widehat{S}(t_i)^2 \sum_{j:t_j \le t_i} \frac{d_j}{n_j(n_j - d_j)},$$

where $\widehat{S}(t_i)$ is the *Kaplan-Meier* estimator, d_j is the number of deaths and n_j is the size of the population at risk during the jth interval. Standard error of *Kaplan-Meier* estimator was given by $\{\widehat{V}[\widehat{S}(t_i)]\}^{1/2}$.

Mean and median lifespan

The Irwin's restricted mean lifespan [2], which was also described by Kaplan and Meier [3], was estimated as the area under the survival curve by the following formula.

$$\widehat{\mu}_\tau = \int_0^\tau \widehat{S}(t)dt,$$

where τ is the largest observed time and $\widehat{S}(t)$ is estimated with the *Kaplan-Meier* method. The median survival time is also commonly used for survival analysis to measure effectiveness of specific treatments or to summarize lifespan data.

$$\widehat{t}_{0.5} = \min(t : \widehat{S}(t) \le 0.5)$$

Variance of mean and median lifespan and confidence interval

The variance of restricted mean lifespan is calculated using the following equation.

$$\widehat{V}[\widehat{\mu}_\tau] = \sum_{i:t_i \le \tau} \left[\int_{t_i}^{\tau} \widehat{S}(t)dt \right]^2 \frac{d_i}{n_i(n_i - d_i)}$$

A 95% confidence interval for the restricted mean is calculated as

$$\widehat{\mu}_\tau \pm 1.96\sqrt{\widehat{V}[\widehat{\mu}_\tau]}$$

We used a 95% confidence interval for median lifespan $\widehat{t}_{0.5}$, based on the linear confidence interval [4]. It takes a set of all time points *t* as a confidence interval that satisfies the following condition.

$$-1.96 \le \frac{\widehat{S}(t) - 0.5}{\widehat{V}^{1/2}[\widehat{S}(t)]} \le 1.96$$

The statistical analysis results using OASIS were identical with those calculated with STATA (Supplementary Table 2).

Lifespan at variable mortality percentages

Mortality is a measure of the death percentage in a given population. Time point at a specific mortality of population can be estimated with a survival function. Generally, survival times at 25%, 50%, 75%, or 90% mortality percentages are compared between different survival data. In the OASIS, Fisher's exact test is provided for statistical comparison at those time points.

Survival and log-cumulative hazard plots

A survival plot shows the relationship between the survival percentage and time, representing the number or population of organisms surviving at each age for a given group. Log

cumulative hazard plot shows changes of mortality rates over specified time period. Mortality rate means the ratio of total deaths in total population over specified period of time. For example, a mortality rate of 11 % in a population of 1,000,000 would mean 1,100 deaths per unit time.

Cox proportional hazards regression

Partial likelihood estimator

OASIS provides two kinds of regression methods using partial likelihood estimator (PLE) and robust estimator through *coxr* function in the R package [5]. The PLE is generally used to estimate the regression coefficients by maximizing the partial likelihood

$$L(\beta) = \prod_{i=1}^{n} \left[\frac{\exp(\beta^{\mathrm{T}} X_i)}{\sum_{t_j \ge t_i} \exp(\beta^{\mathrm{T}} X_j)} \right]^{\delta_i}, \delta_i = \begin{cases} 1 & \text{for the deaths observed} \\ 0 & \text{otherwise} \end{cases},$$

where β is a k × 1 vector of regression coefficients, X_i is a k × 1 vector of risk factors, and t_i is a observed time of event. To estimate β, which maximizes the partial likelihood, Cox's estimator solves the following score function that is the derivative with respect to β of the log partial likelihood function.

$$\sum_{i=1}^{n} \left[X_i - \frac{\sum_{t_j \ge t_i} X_j \exp(\beta^{\mathrm{T}} X_j)}{\sum_{t_j \ge t_i} \exp(\beta^{\mathrm{T}} X_j)} \right] \delta_i = 0,$$

where t_i is a observed time of event and δ_i equals 1 for the deaths observed and 0 otherwise.

Robust estimator

While the PLE is commonly used for Cox proportional hazards regression, it is known to be sensitive to outliers and the deflection of underlying assumption [6]. Especially the PLE method is strongly influenced by large values of $t\ exp(\beta^T X)$. To reduce the influence of large values of $t\ exp(\beta^T X)$, Minder and Bednarski introduced smooth modification to the score function of the partial likelihood method [6],

$$\sum_{i=1}^{n} A(t_i, X_i) \left[X_i - \frac{\sum_{t_j \ge t_i} A(t_i, X_j) X_j \exp(\beta^{\mathrm{T}} X_j)}{\sum_{t_j \ge t_i} A(t_i, X_j) \exp(\beta^{\mathrm{T}} X_j)} \right] \delta_i = 0 \,,$$

where smooth function $A(t_i, X_i)$ is defined as $A(t_i, X_i) = M - min(\ M, t_i\ exp(\beta^T X_i)\ )$ and M is a 95th percentile of the samples [$t_1\ exp(\beta^T X_1)$, …, $t_n\ exp(\beta^T X_n)$]. The smooth functions in the outer and inside sum have an reducing effect on large values of $t\ exp(\beta^T X)$ and $\beta^T X$ respectively. OASIS adopted *coxr* function implemented in the R packages [5] to provide robust estimation. In the *coxr* function, a better solution for smooth function $A(t_i, X_i)$ is used by using $\Lambda(t)exp(\beta^T X)$ instead of heuristic $t\ exp(\beta^T X_i)$, where $\Lambda(t)$ is -log$S(t)$.

Supplementary References

1. Jewell NP, Lei X, Ghani AC, Donnelly CA, Leung GM, et al. (2007) Non-parametric estimation of the case fatality ratio with competing risks data: an application to Severe Acute Respiratory Syndrome (SARS). Stat Med 26: 1982-1998.
2. Irwin JO (1949) The standard error of an estimate of expectation of life, with special reference to expectation of tumourless life in experiments with mice. J Hyg (Lond) 47: 188.
3. Kaplan EL, Meier P (1958) Nonparametric estimation from incomplete observations. Journal of the American Statistical Association 53: 457-481.
4. Brookmeyer R, Crowley J (1982) A confidence interval for the median survival time. . Biometrics 38: 29-41.
5. R Development Core Team and contributors worldwide, 2008.
6. Minder CE, Bednarski T (1996) A robust method for proportional hazards regression. Stat Med 15: 1033-1047.
