# Supplementary material for: OASIS: Online Application for the Survival Analysis of Lifespan Assays Performed in Aging Research
Source: PLoS One. 2011 Aug 15;6(8):e23525. doi: 10.1371/journal.pone.0023525 (PMC3156233; doi:10.1371/journal.pone.0023525)
Supplement: Table S1 — Statistical methods used for survival analyses (PDF) [file pone.0023525.s002.pdf]

Table S1. Statistical methods used for survival analyses

Index	Paper	Graph		Statistical Test							Lifespan Statistics					
		Survival Curve	Log Cumulative Hazard Plot or Mortality Curve	Log Rank Test	Wilcoxon Rank-Sum Test	Fisher's Exact Test	Cox's test	T-Test	Gompertz	Mann-Whitney Test	Maximum Lifespan	Mean Lifespan	Median Lifespan	Last quartile lifespan	Intercept mortality rate function	Slope mortality rate function
1	**Alcedo and Kenyon, 2004**	O		O								O		O		
2	**Andrade et al., 2005**					O										
3	**Bauer et al., 2007**	O		O							O	O	O			
4	**Bishop and Guarente, 2007**	O		O								O				
5	**Bluher et al., 2003**	O		O							O		O			
6	**Budovskaya et al., 2008**	O		O												
7	**Civetta and Clark, 2000**		O									O			O	O
8	**Clancy et al., 2001**	O		O								O	O			
9	**Cohen et al., 2006**	O										O				
10	**Cristina et al., 2009**	O		O								O				
11	**Curran and Ruvkun, 2007**			O								O	O			
12	**Doonan et al., 2008**	O		O								O				
13	**Evason et al., 2005**	O									O	O				
14	**Fabrizio et al., 2005**	O						O		O		O				
15	**Fox et al., 2004**	O	O									O				O
16	**Gallagher et al., 2000**					O										
17	**Gerisch et al., 2001**	O						O		O	O	O		O		
18	**Gerisch et al., 2007**	O		O							O	O				
19	**Greer et al., 2007**	O	O	O								O	O			
20	**Hansen et al., 2007**	O		O							O	O				
21	**Hansen et al., 2008**	O		O								O				
22	**Harrison et al., 2009**	O		O		O					O	O	O			
23	**Holzenberger et al., 2003**	O		O			O	O				O				
24	**Honjoh et al., 2009**	O		O				O				O				
25	**Hwangbo et al., 2004**	O											O			
26	**Joeng et al., 2004**	O		O							O	O				
27	**Kapahi et al., 2004**	O		O								O				
28	**Lambert et al., 2004**	O		O		O										
29	**Li et al., 2008**	O		O								O				
30	**Libert et al., 2007**	O	O	O								O	O			
31	**Liu et al., 2005**	O		O							O	O	O			
32	**Mair et al., 2003**		O						O							O
33	**Matheu et al., 2007**	O		O							O		O			
34	**Mehta et al., 2009**	O			O							O				
35	**Morley and Morimoto, 2004**	O										O				
36	**Olsen et al., 2006**	O														
37	**Padmanabhan et al., 2009**	O										O				
38	**Petrascheck et al., 2007**	O		O							O	O				
39	**Ren et al., 2007**	O		O									O			
40	**Schriner et al., 2005**	O									O	O	O			
41	**Shaw et al., 2007**	O										O				
42	**Sheridan et al., 2000**					O										
43	**Smith et al., 2008**	O			O											
44	**Steffen et al., 2008**	O			O						O	O				
45	**Syntichaki et al., 2007**	O														
46	**Tong et al., 2007**	O		O							O	O	O			
47	**Valenzano et al., 2006**	O	O	O							O		O		O	O
48	**Viswanathan et al., 2005**	O		O							O	O				
49	**Wang et al., 2005**	O		O								O				
50	**Wilson et al., 2006**	O		O							O	O				
51	**Wood et al., 2004**	O	O	O								O	O			
52	**Wu et al., 2009**	O	O						O		O	O				
53	**Xue et al., 2007**	O		O												

References

1 Alcedo, J. and Kenyon, C., 2004. Regulation of *C. elegans* longevity by specific gustatory and olfactory neurons. Neuron 41, 45-55.

2 Andrade, M.C., Gu, L. and Stoltz, J.A., 2005. Novel male trait prolongs survival in suicidal mating. Biol Lett 1, 276-279.

3 Bauer, J.H., Chang, C., Morris, S.N., Hozier, S., Andersen, S., Waitzman, J.S. and Helfand, S.L., 2007. Expression of dominant-negative Dmp53 in the adult *fly* brain inhibits insulin signaling. Proc Natl Acad Sci U S A 104, 13355-13360.

4 Bishop, N.A. and Guarente, L., 2007. Two neurons mediate diet-restriction-induced longevity in *C. elegans* . Nature 447, 545-549.

5 Bluher, M., Kahn, B.B. and Kahn, C.R., 2003. Extended longevity in *mice* lacking the insulin receptor in adipose tissue. Science 299, 572-574.

6 Budovskaya, Y.V., Wu, K., Southworth, L.K., Jiang, M., Tedesco, P., Johnson, T.E. and Kim, S.K., 2008. An elt-3/elt-5/elt-6 GATA transcription circuit guides aging in *C. elegans* . Cell 134, 291-303.

7 Civetta, A. and Clark, A.G., 2000. Correlated effects of sperm competition and postmating female mortality. Proc Natl Acad Sci U S A 97, 13162-13165.

8 Clancy, D.J., Gems, D., Harshman, L.G., Oldham, S., Stocker, H., Hafen, E., Leevers, S.J. and Partridge, L., 2001. Extension of life-span by loss of CHICO, a *Drosophila* insulin receptor substrate protein. Science 292, 104-106.

9 Cohen, E., Bieschke, J., Perciavalle, R.M., Kelly, J.W. and Dillin, A., 2006. Opposing activities protect against age-onset proteotoxicity. Science 313, 1604-1610.

10 Cristina, D., Cary, M., Lunceford, A., Clarke, C. and Kenyon, C., 2009. A regulated response to impaired respiration slows behavioral rates and increases lifespan in *Caenorhabditis elegans* . PLoS Genet 5, e1000450.

11 Curran, S.P. and Ruvkun, G., 2007. Lifespan regulation by evolutionarily conserved genes essential for viability. PLoS Genet 3, e56.

12 Doonan, R., McElwee, J.J., Matthijssens, F., Walker, G.A., Houthoofd, K., Back, P., Matscheski, A., Vanfleteren, J.R. and Gems, D., 2008. Against the oxidative damage theory of aging: superoxide dismutases protect against oxidative stress but have little or no effect on life span in *Caenorhabditis elegans* . Genes Dev 22, 3236-3241.

13 Evason, K., Huang, C., Yamben, I., Covey, D.F. and Kornfeld, K., 2005. Anticonvulsant medications extend worm life-span. Science 307, 258-262.

14 Fabrizio, P., Gattazzo, C., Battistella, L., Wei, M., Cheng, C., McGrew, K. and Longo, V.D., 2005. Sir2 blocks extreme life-span extension. Cell 123, 655-667.

15 Fox, C.W., Bush, M.L., Roff, D.A. and Wallin, W.G., 2004. Evolutionary genetics of lifespan and mortality rates in two populations of the seed beetle, *Callosobruchus maculatus* . Heredity 92, 170-181.

16 Gallagher, I.M., Jenner, P., Glover, V. and Clow, A., 2000. CuZn-superoxide dismutase transgenic *mice* : no effect on longevity, locomotor activity and 3H-mazindol and 3H-spiperone binding over 19 months. Neurosci Lett 289, 221-223.

17 Gerisch, B., Rottiers, V., Li, D., Motola, D.L., Cummins, C.L., Lehrach, H., Mangelsdorf, D.J. and Antebi, A., 2007. A bile acid-like steroid modulates *Caenorhabditis elegans* lifespan through nuclear receptor signaling. Proc Natl Acad Sci U S A 104, 5014-5019.

18 Gerisch, B., Weitzel, C., Kober-Eisermann, C., Rottiers, V. and Antebi, A., 2001. A hormonal signaling pathway influencing *C. elegans* metabolism, reproductive development, and life span. Dev Cell 1, 841-851.

19 Greer, E.L., Dowlatshahi, D., Banko, M.R., Villen, J., Hoang, K., Blanchard, D., Gygi, S.P. and Brunet, A., 2007. An AMPK-FOXO pathway mediates longevity induced by a novel method of dietary restriction in *C. elegans* . Curr Biol 17, 1646-1656.

20 Hansen, M., Chandra, A., Mitic, L.L., Onken, B., Driscoll, M. and Kenyon, C., 2008. A role for autophagy in the extension of lifespan by dietary restriction in *C. elegans* . PLoS Genet 4, e24.

21 Hansen, M., Taubert, S., Crawford, D., Libina, N., Lee, S.J. and Kenyon, C., 2007. Lifespan extension by conditions that inhibit translation in *Caenorhabditis elegans* . Aging Cell 6, 95-110.

22 Harrison, D.E., Strong, R., Sharp, Z.D., Nelson, J.F., Astle, C.M., Flurkey, K., Nadon, N.L., Wilkinson, J.E., Frenkel, K., Carter, C.S., Pahor, M., Javors, M.A., Fernandez, E. and Miller, R.A., 2009. Rapamycin fed late in life extends lifespan in genetically heterogeneous *mice* . Nature 460, 392-395.

23 Holzenberger, M., Dupont, J., Ducos, B., Leneuve, P., Geloen, A., Even, P.C., Cervera, P. and Le Bouc, Y., 2003. IGF-1 receptor regulates lifespan and resistance to oxidative stress in *mice*. Nature 421, 182-187.
24 Honjoh, S., Yamamoto, T., Uno, M. and Nishida, E., 2009. Signalling through RHEB-1 mediates intermittent fasting-induced longevity in *C. elegans*. Nature 457, 726-730.
25 Hwangbo, D.S., Gershman, B., Tu, M.P., Palmer, M. and Tatar, M., 2004. *Drosophila* dFOXO controls lifespan and regulates insulin signalling in brain and fat body. Nature 429, 562-566.
26 Joeng, K.S., Song, E.J., Lee, K.J. and Lee, J., 2004. Long lifespan in worms with long telomeric DNA. Nat Genet 36, 607-611.
27 Kapahi, P., Zid, B.M., Harper, T., Koslover, D., Sapin, V. and Benzer, S., 2004. Regulation of lifespan in *Drosophila* by modulation of genes in the TOR signaling pathway. Curr Biol 14, 885-890.
28 Lambert, H.E., Gregory, W.M., Nelstrop, A.E. and Rustin, G.J., 2004. Long-term survival in 463 women treated with platinum analogs for advanced epithelial carcinoma of the ovary: life expectancy compared to women of an age-matched normal population. Int J Gynecol Cancer 14, 772-778.
29 Li, J., Ebata, A., Dong, Y., Rizki, G., Iwata, T. and Lee, S.S., 2008. *Caenorhabditis elegans* HCF-1 functions in longevity maintenance as a DAF-16 regulator. PLoS Biol 6, e233.
30 Libert, S., Zwiener, J., Chu, X., Vanvoorhies, W., Roman, G. and Pletcher, S.D., 2007. Regulation of *Drosophila* life span by olfaction and food-derived odors. Science 315, 1133-1137.
31 Liu, X., Jiang, N., Hughes, B., Bigras, E., Shoubridge, E. and Hekimi, S., 2005. Evolutionary conservation of the clk-1-dependent mechanism of longevity: loss of mclk1 increases cellular fitness and lifespan in *mice*. Genes Dev 19, 2424-2434.
32 Mair, W., Goymer, P., Pletcher, S.D. and Partridge, L., 2003. Demography of dietary restriction and death in *Drosophila*. Science 301, 1731-1733.
33 Matheu, A., Maraver, A., Klatt, P., Flores, I., Garcia-Cao, I., Borras, C., Flores, J.M., Vina, J., Blasco, M.A. and Serrano, M., 2007. Delayed ageing through damage protection by the Arf/p53 pathway. Nature 448, 375-379.
34 Mehta, R., Steinkraus, K.A., Sutphin, G.L., Ramos, F.J., Shamieh, L.S., Huh, A., Davis, C., Chandler-Brown, D. and Kaeberlein, M., 2009. Proteasomal regulation of the hypoxic response modulates aging in *C. elegans*. Science 324, 1196-1198.
35 Morley, J.F. and Morimoto, R.I., 2004. Regulation of longevity in *Caenorhabditis elegans* by heat shock factor and molecular chaperones. Mol Biol Cell 15, 657-664.
36 Olsen, A., Vantipalli, M.C. and Lithgow, G.J., 2006. Checkpoint proteins control survival of the postmitotic cells in *Caenorhabditis elegans*. Science 312, 1381-1385.
37 Padmanabhan, S., Mukhopadhyay, A., Narasimhan, S.D., Tesz, G., Czech, M.P. and Tissenbaum, H.A., 2009. A PP2A regulatory subunit regulates *C. elegans* insulin/IGF-1 signaling by modulating AKT-1 phosphorylation. Cell 136, 939-951.
38 Petrascheck, M., Ye, X. and Buck, L.B., 2007. An antidepressant that extends lifespan in adult *Caenorhabditis elegans*. Nature 450, 553-556.
39 Ren, C., Webster, P., Finkel, S.E. and Tower, J., 2007. Increased internal and external bacterial load during *Drosophila* aging without life-span trade-off. Cell Metab 6, 144-152.
40 Schriner, S.E., Linford, N.J., Martin, G.M., Treuting, P., Ogburn, C.E., Emond, M., Coskun, P.E., Ladiges, W., Wolf, N., Van Remmen, H., Wallace, D.C. and Rabinovitch, P.S., 2005. Extension of murine life span by overexpression of catalase targeted to mitochondria. Science 308, 1909-1911.
41 Shaw, W.M., Luo, S., Landis, J., Ashraf, J. and Murphy, C.T., 2007. The *C. elegans* TGF-beta Dauer pathway regulates longevity via insulin signaling. Curr Biol 17, 1635-1645.
42 Sheridan, R.L., Remensnyder, J.P., Schnitzer, J.J., Schulz, J.T., Ryan, C.M. and Tompkins, R.G., 2000. Current expectations for survival in pediatric burns. Arch Pediatr Adolesc Med 154, 245-249.
43 Smith, E.D., Tsuchiya, M., Fox, L.A., Dang, N., Hu, D., Kerr, E.O., Johnston, E.D., Tchao, B.N., Pak, D.N., Welton, K.L., Promislow, D.E., Thomas, J.H., Kaeberlein, M. and Kennedy, B.K., 2008. Quantitative evidence for conserved longevity pathways between divergent eukaryotic species. Genome Res 18, 564-570.
44 Steffen, K.K., MacKay, V.L., Kerr, E.O., Tsuchiya, M., Hu, D., Fox, L.A., Dang, N., Johnston, E.D., Oakes, J.A., Tchao, B.N., Pak, D.N., Fields, S., Kennedy, B.K. and Kaeberlein, M., 2008. *Yeast* life span extension by depletion of 60s ribosomal subunits is mediated by Gcn4. Cell 133, 292-302.
45 Syntichaki, P., Troulinaki, K. and Tavernarakis, N., 2007. eIF4E function in somatic cells modulates ageing in *Caenorhabditis elegans*. Nature 445, 922-926.
46 Tong, J.J., Schriner, S.E., McCleary, D., Day, B.J. and Wallace, D.C., 2007. Life extension through neurofibromin mitochondrial regulation and antioxidant therapy for neurofibromatosis-1 in *Drosophila* melanogaster. Nat Genet 39, 476-485.
47 Valenzano, D.R., Terzibasi, E., Genade, T., Cattaneo, A., Domenici, L. and Cellerino, A., 2006. Resveratrol prolongs lifespan and retards the onset of age-related markers in a short-lived vertebrate. Curr Biol 16, 296-300.
48 Viswanathan, M., Kim, S.K., Berdichevsky, A. and Guarente, L., 2005. A role for SIR-2.1 regulation of ER stress response genes in determining *C. elegans* life span. Dev Cell 9, 605-615.
49 Wang, M.C., Bohmann, D. and Jasper, H., 2005. JNK extends life span and limits growth by antagonizing cellular and organism-wide responses to insulin signaling. Cell 121, 115-125.
50 Wilson, M.A., Shukitt-Hale, B., Kalt, W., Ingram, D.K., Joseph, J.A. and Wolkow, C.A., 2006. Blueberry polyphenols increase lifespan and thermotolerance in *Caenorhabditis elegans*. Aging Cell 5, 59-68.
51 Wood, J.G., Rogina, B., Lavu, S., Howitz, K., Helfand, S.L., Tatar, M. and Sinclair, D., 2004. Sirtuin activators mimic caloric restriction and delay ageing in metazoans. Nature 430, 686-689.
52 Wu, D., Cypser, J.R., Yashin, A.I. and Johnson, T.E., 2009. Multiple mild heat-shocks decrease the Gompertz component of mortality in *Caenorhabditis elegans*. Exp Gerontol
53 Xue, H., Xian, B., Dong, D., Xia, K., Zhu, S., Zhang, Z., Hou, L., Zhang, Q., Zhang, Y. and Han, J.D., 2007. A modular network model of aging. Mol Syst Biol 3, 147.
