# Supplementary material for: OASIS: Online Application for the Survival Analysis of Lifespan Assays Performed in Aging Research
Source: PLoS One. 2011 Aug 15;6(8):e23525. doi: 10.1371/journal.pone.0023525 (PMC3156233; doi:10.1371/journal.pone.0023525)
Supplement: Table S2 — Comparisons of OASIS results with reference data (PDF) [file pone.0023525.s003.pdf]

Table S2. Comparisons of OASIS results with reference data

Data 1

URL http://www.weibull.com/LifeDataWeb/nonparametric_analysis.htm

Solution to Additional Reliability Analysis Tools Example 8

OASIS Result										Reference Data		
Time	At Risk	Dead	Censored	Percent Mortality(%)	S_hat	Var S_hat	SE(S)	Surv_Time	Time	Dead	S_hat	
9	21	3	1	14.29%	0.85714	0.00583	0.07636	9	9	3	0.857143	
11	17	1	0	19.33%	0.80672	0.00756	0.08694	10.71429	11	1	0.806723	
12	16	0	1	19.33%	0.80672	0.00756	0.08694	11.52101	12	0	0.806723	
13	15	1	1	24.71%	0.75294	0.00928	0.09635	12.32773	13	1	0.752941	
15	13	0	1	24.71%	0.75294	0.00928	0.09635	13.83361	15	0	0.752941	
17	12	1	0	30.98%	0.6902	0.01141	0.10681	15.3395	17	1	0.690196	
21	11	1	0	37.25%	0.62745	0.01301	0.11405	18.10028	21	1	0.627451	
22	10	0	1	37.25%	0.62745	0.01301	0.11405	18.72773	22	0	0.627451	
24	9	0	1	37.25%	0.62745	0.01301	0.11405	19.98263	24	0	0.627451	
26	8	0	1	37.25%	0.62745	0.01301	0.11405	21.23754	26	0	0.627451	
28	7	1	0	46.22%	0.53782	0.01644	0.12823	22.49244	28	1	0.537815	
30	6	1	0	55.18%	0.44818	0.01811	0.13459	23.56807	30	1	0.488179	
32	5	0	1	55.18%	0.44818	0.01811	0.13459	24.46443	32	0	0.488179	
35	4	0	2	55.18%	0.44818	0.01811	0.13459	25.80896	35	0	0.488179	
39	2	0	1	55.18%	0.44818	0.01811	0.13459	27.60168	39	0	0.488179	
41	1	0	1	55.18%	0.44818	0.01811	0.13459	28.49804	41	0	0.488179	

Data 2

URL http://sbi.postech.ac.kr/oasis/introduction.html

daf-2(e1370) and wild type treated with control or *daf-16* RNAi (Example in OASIS web site)

OASIS Result						STATA Result					
Strin	No. of subjects	mean	Std. Err.	[95% Conf.	Interval]	strain	subjects	mean	Std. Err.	[95% Conf.	Interval]
WT	52	19.35	1.07	17.24	21.45	WT	52	19.34552	1.074908	17.2387	21.4523
daf-2(e1370)	223	32.87	0.64	31.63	34.12	*daf-2(e1370)*	223	32.87417	0.636561	31.6265	34.1218
daf-16(RNAi)	127	17.74	0.43	16.91	18.57	*daf-16(RNAi)*	127	17.7403	0.4258734	16.9056	18.575
daf-16(RNAi); daf-2(e1370)	366	20.84	0.46	19.94	21.73	*daf-16(RNAi); daf-2(e1370)*	366	20.83829	0.4564434	19.9437	21.7329

OASIS Result				STATA Result			
		Log-rank test				Log-rank test	
Type	Name	Chi2	Pr > Chi2	Type	Name	Chi2	Pr > Chi2
Control	WT			Control	WT		
	daf-2(e1370)	119.19	0.00E+00		*daf-2(e1370)*	119.19	0.00E+00
	daf-16(RNAi)	5.03	2.50E-02		*daf-16(RNAi)*	5.03	2.50E-02
	daf-16(RNAi); daf-2(e137	2.51	1.13E-01		*daf-16(RNAi); daf-2(e13*	2.51	1.13E-01
Control	*daf-2(e1370)*			Control	*daf-2(e1370)*		
	WT	119.19	0.00E+00		WT	119.19	0.00E+00
	daf-16(RNAi)	206.82	0.00E+00		*daf-16(RNAi)*	206.82	0.00E+00
	daf-16(RNAi); daf-2(e137	146.07	0.00E+00		*daf-16(RNAi); daf-2(e13*	146.07	0.00E+00
Control	*daf-16(RNAi)*			Control	*daf-16(RNAi)*		
	WT	5.03	2.50E-02		WT	5.03	2.50E-02
	daf-2(e1370)	206.82	0.00E+00		*daf-2(e1370)*	206.82	0.00E+00
	daf-16(RNAi); daf-2(e137	21.38	3.76E-06		*daf-16(RNAi); daf-2(e13*	21.38	0.00E+00
Control	*f-16(RNAi); daf-2(e137*			Control	*16(RNAi); daf-2(e1370)*		
	WT	2.51	1.13E-01		WT	2.51	1.13E-01
	daf-2(e1370)	146.07	0.00E+00		*daf-2(e1370)*	146.07	0.00E+00
	daf-16(RNAi)	21.38	3.76E-06		*daf-16(RNAi)*	21.38	0.00E+00

Data 3

URL http://www.statsdirect.com/help/survival_analysis/kaplan.htm

Example of test workbook (Survival worksheet: Group Surv, Time Surv, Censor Surv)

Group 1

OASIS Result									Reference Data					
Time	At Risk	Dead	Censored	Percent Mortality(%)	S_hat	Var S_hat	SE(S)	Surv_Time	Time	At risk	Dead	Censored	S	SE(S)
142	22	1	0	4.55%	0.95455	0.00197	0.04441	142	142	22	1	0	0.954545	0.044409
157	21	1	0	9.09%	0.90909	0.00376	0.06129	156.31818	157	21	1	0	0.909091	0.061291
163	20	1	0	13.64%	0.86364	0.00535	0.07317	161.77273	163	20	1	0	0.863636	0.073165
198	19	1	0	18.18%	0.81818	0.00676	0.08223	192	198	19	1	0	0.818182	0.08223
204	18	0	1	18.18%	0.81818	0.00676	0.08223	196.90909	204	18	0	1	0.818182	0.08223
205	17	1	0	22.99%	0.77005	0.00817	0.09039	197.72727	205	17	1	0	0.770053	0.090387
232	16	3	0	37.43%	0.62567	0.01104	0.10507	218.51872	232	16	3	0	0.625668	0.105069
233	13	4	0	56.68%	0.43316	0.01171	0.10819	219.14439	233	13	4	0	0.433155	0.108192
239	9	1	0	61.50%	0.38503	0.01131	0.10634	221.74332	239	9	1	0	0.385027	0.106338
240	8	1	0	66.31%	0.3369	0.01068	0.10337	222.12834	240	8	1	0	0.336898	0.103365
261	7	1	0	71.12%	0.28877	0.00984	0.09917	229.20321	261	7	1	0	0.28877	0.099172
280	6	2	0	80.75%	0.19251	0.00746	0.08637	234.68984	280	6	2	0	0.192513	0.086369
295	4	2	0	90.37%	0.09626	0.00418	0.06466	237.57754	295	4	2	0	0.096257	0.064663
323	2	1	0	95.19%	0.04813	0.0022	0.04694	240.27273	323	2	1	0	0.048128	0.046941
344	1	0	1	95.19%	0.04813	0.0022	0.04694	241.28342	344	1	0	1	0.048128	0.046941

OASIS Result			Reference Data		
Median Survival Time	[Brookmeyer-Crowley 95% Conf.	Interval]	Median Survival Time	[Brookmeyer-Crowley 95% Conf.	Interval]
233	232	240	233	232	240

Group 2

OASIS Result									Reference Data					
Time	At Risk	Dead	Censored	Percent Mortality(%)	S_hat	Var S_hat	SE(S)	Surv_Time	Time	At risk	Dead	Censored	S	SE(S)
143	19	1	0	5.26%	0.94737	0.00262	0.05123	143	143	19	1	0	0.947368	0.051228
165	18	1	0	10.53%	0.89474	0.00496	0.07041	163.84211	165	18	1	0	0.894737	0.070406
188	17	2	0	21.05%	0.78947	0.00875	0.09353	184.42105	188	17	2	0	0.789474	0.093529
190	15	1	0	26.32%	0.73684	0.01021	0.10102	186	190	15	1	0	0.736842	0.101023
192	14	1	0	31.58%	0.68421	0.01137	0.10664	187.47368	192	14	1	0	0.684211	0.106639
206	13	1	0	36.84%	0.63158	0.01225	0.11066	197.05263	206	13	1	0	0.631579	0.110665
208	12	1	0	42.11%	0.57895	0.01283	0.11327	198.31579	208	12	1	0	0.578947	0.113269
212	11	1	0	47.37%	0.52632	0.01312	0.11455	200.63158	212	11	1	0	0.526316	0.114549
216	10	1	1	52.63%	0.47368	0.01312	0.11455	202.73684	216	10	1	1	0.473684	0.114549
220	8	1	0	58.55%	0.41447	0.01311	0.11452	204.63158	220	8	1	0	0.414474	0.114515
227	7	1	0	64.47%	0.35526	0.01264	0.11243	207.53289	227	7	1	0	0.355263	0.112426
230	6	1	0	70.39%	0.29605	0.0117	0.10816	208.59868	230	6	1	0	0.296053	0.108162
235	5	1	0	76.32%	0.23684	0.01029	0.10145	210.07895	235	5	1	0	0.236842	0.10145
244	4	0	1	76.32%	0.23684	0.01029	0.10145	212.21053	244	4	0	1	0.236842	0.10145
246	3	1	0	84.21%	0.15789	0.00873	0.09343	212.68421	246	3	1	0	0.157895	0.093431
265	2	1	0	92.11%	0.07895	0.0053	0.07279	215.68421	265	2	1	0	0.078947	0.072792
303	1	1	0	100.00%	0	0	0	218.68421	303	1	1	0	0	*

OASIS Result			Reference Data		
Median Survival Time	[Brookmeyer-Crowley 95% Conf.	Interval]	Median Survival Time	[Brookmeyer-Crowley 95% Conf.	Interval]
216	192	230	216	192	230
