# Supplementary material for: OASIS: Online Application for the Survival Analysis of Lifespan Assays Performed in Aging Research
Source: PLoS One. 2011 Aug 15;6(8):e23525. doi: 10.1371/journal.pone.0023525 (PMC3156233; doi:10.1371/journal.pone.0023525)
Supplement: Table S3 — Web-based statistical methods for survival analysis (PDF) [file pone.0023525.s004.pdf]

Table S3. Web-based statistical methods for survival analysis

Description		URL
Statistical Test	Wilcoxon signed-rank test	http://faculty.vassar.edu/lowry/wilcoxon.html
	Wilcoxon rank sum test	http://elegans.swmed.edu/~leon/stats/utest.html
	Fisher's exact test	http://www.socr.ucla.edu/htmls/ana/FishersExactTest_Analysis.html
	Fisher's exact test	http://faculty.vassar.edu/lowry/fisher.html
	Fisher's exact test	http://www.langsrud.com/fisher.htm
Lifespan Statistics	Log Rank Test	http://bioinf.wehi.edu.au/software/russell/logrank/index.html
	Kaplan-Meier survival function	http://www.hutchon.net/Kaplan-Meier.htm
	SURVSOFT	http://www.krebsregister-bayern.de/software_e.html
